# Risk factors associated with length of hospital stay and medical expenses in pulmonary abscess patients: retrospective study

**DOI:** 10.7717/peerj.15106

**Published:** 2023-04-12

**Authors:** Tianyi Zhu, Wei Yang, Wei Lu

**Affiliations:** Department of respiratory, General Hospital of Northern Theater Command, Shenyang, China

**Keywords:** Pulmonary abscess, Length of hospital stay, Medical expenses, Hospitalization

## Abstract

**Background:**

Pulmonary abscess carries a high mortality and requires long-term managements. A better understanding of the risk factors associated with the prolonged hospital stay and high medical expenses in these patients can improve the management strategy in individual patient and optimize the overall healthcare resources.

**Methods:**

We performed a retrospective study and reviewed the medical records on consecutive patients hospitalized at the Department of Respiratory Medicine of the General Hospital of Northern Theater Command, Shenyang, Liaoning, China, between January 1, 2015, and December 31, 2020. Demographics, comorbidity, clinical symptoms, laboratory tests, length of hospital stay, and medical expenses were recorded. Their relationships with the length of hospital stay and medical expenses in pulmonary abscess patients were analyzed.

**Results:**

There were 190 patients with the pulmonary abscess and 12,189 patients without the pulmonary abscess. Compared with patients without the pulmonary abscess, patients with the pulmonary abscess had longer hospital stays (21.8 ± SD *vs* 12.8 ± SD, *P* < 0.01), In patients with the pulmonary abscess, the mean length of hospital stay was 5.3 days longer in male *vs* female patients (*P* = 0.025). Multivariate linear regression analyses showed that extrapulmonary disease and clinical symptoms were associated with the length of hospital stay and medical expenses, respectively. In addition, anemia was associated with both the length of hospital stay and medical expenses. Sex and hypoproteinemia were associated with the medical expenses.

**Conclusions:**

The mean length of hospital stay was longer in patients with the pulmonary abscess than those without the pulmonary abscess. The length of hospital stay and medical expense were associated with sex, clinical symptoms, extrapulmonary disease, and abnormal laboratory tests in patients with the pulmonary abscess.

## Introduction

A pulmonary abscess is a severe pulmonary infection due to various pathogens (Duncan et al., 2017; Kuhajda et al., 2015; Sabbula, Rammohan & Akella, 2022). Its common clinical presentations include fever, cough, expectoration, chest pain, and dyspnea. The diagnosis of a pulmonary abscess is based on the clinical symptoms, laboratory tests, and pulmonary imaging (Feki et al., 2019; Hecker et al., 2012). Appropriate and timely antibiotic treatments (Tan et al., 2020; Zhang et al., 2021) together adequate drainage are critical for the disease control (Abu-Awwad et al., 2015; Kelogrigoris et al., 2011; Lee et al., 2021; Liu, Wang & Xu, 2019; Marra, Hillejan & Ukena, 2015; Wali, 2012).

The incidence of pulmonary abscess has reduced at the end of the 20^th^ century from improved medical conditions and the oral hygiene among the general population. However, since the beginning of the 21^st^ century, the incidence of pulmonary abscess has rebounded, due to the increased antibiotic resistance, the aging population, and a high prevalence of cardiovascular and cerebrovascular diseases, diabetes, and cancer among the population, which pose a great challenge on the control of this disease (Cai et al., 2019; Dyrhovden et al., 2019; Patradoon-Ho & Fitzgerald, 2007). The presence of a pulmonary abscess has been associated with a high mortality, a long treatment cycle, and huge management costs. Although many studies have explored pulmonary abscess patients from the perspectives of anti-infective treatment, drug sensitivity of infectious pathogens, drainage methods, *etc*. (Hou et al., 2020; Hu, 2020; Mohapatra, Rajaram & Mallick, 2018), there were limited studies on the length of hospital stay and medical expenses on these patients. In general, the length of hospital stay can be influenced by many factors, such as baseline demographics, underlying comorbidities, disease complications, and medical expenses (Chen et al., 2017; Liu et al., 2021). Prolonged length of hospital stay has been associated with high medical expenses, cognitive impairment, functional dependence, and high comorbidity (Bo et al., 2016; Kamitani et al., 2020). Understanding the risk factors associated with the prolonged length of hospital stay and high medical expenses in patients with pulmonary abscess can improve the management of individual patients and optimize the overall healthcare resources.

In the current study, we hypothesized that certain factors were associated with prolonged length of hospital stay and high medical expenses in patients with pulmonary abscess. We performed a retrospective study to identify these risk factors in order to improve the healthcare and overall resource utilization.

## Materials and Methods

### Study design and participant selection

We performed a retrospective study and reviewed the medical records on consecutive patients who were hospitalized in the Department of Respiratory Medicine of the General Hospital of Northern Theater Command, Shenyang, Liaoning, China, during the past 5 years (January 1, 2015, to December 31, 2020).

All hospitalized patients during the study period were reviewed. Those patients with incomplete medical records were excluded.

### Data collections

Medical records were reviewed and data, including sex, age, comorbidity, clinical symptoms, length of hospital stay, and medical expenses during the hospitalization, were collected. Medical expenses were the entire cost of medical managements during the hospitalization and were calculated based on the hospital discharge settlement bills. The diagnosis of pulmonary abscess was based on the previous published article (Sabbula, Rammohan & Akella, 2022).

### Statistical analysis

All of the statistical analyses were performed in SPSS 17.0 (SPSS, IBM, New York, USA). The continuous data are presented as mean ± standard deviation and compared by the *t*-test and one-way analysis of variance (ANOVA). The categorical data are presented as numbers with percentages and compared by chi-square test. Bivariate analyses were performed by the Pearson correlation analysis or chi-square test. Scatter plots were created to show the correlations between age with the length of hospital stay and medical expenses Multivariate linear regression analyses were performed to study the relationships between various factors and the length of hospital stay and medical expenses in patients with pulmonary abscess. *P* < 0.05 was considered statistically significant.

### Ethical consideration

The study protocol was approved by the hospital ethics committee [Y (2022) 010]. The entire research was performed in accordance with the relevant guidelines and regulations. Informed consent was waived due to the retrospective design of the study.

## Results

### Baseline characteristics of hospitalized patients

A total of 12,364 inpatients were reviewed during the study period. There were 190 patients with the pulmonary abscess and 12,174 patients without the pulmonary abscess.

### Comparisons between inpatients with and without pulmonary abscess

As shown in Table 1, compared with the patients without the pulmonary abscess, the patients with the pulmonary abscess were more likely to be younger, male, and without baseline medical conditions. However, the patients with the pulmonary abscess had a statistically significantly longer hospital stay than the patients without the patients without the pulmonary abscess (*P* < 0.01). The patients with the pulmonary abscess also had a higher medical expenses than the patients without the pulmonary abscess, although the difference did not reach a statistical significance (*P* = 0.55).

**Table 1:** Clinical characteristic comparisons between patients with or without pulmonary abscess.

Variables	Patients with pulmonary abscess (*N* = 190)	Patients without pulmonary abscess (*N* = 12,174)	*P*
Age, years, M ± SD	60.1 ± 15.2	63.7 ± 15.9	<0.01
Sex, N (%)			
Male	142 (74.7%)	7,775 (63.9%)	<0.01
Female	48 (25.3%)	4,399 (36.1%)	
Baseline medical conditions, N (%)			
Yes	114 (60.0%)	8,260 (67.9%)	0.02
Underlying extrapulmonary disease, N (%)			
Yes	83 (43.7%)	6,131 (50.4%)	0.07
Diabetes	49 (25.8%)	1,919 (15.8%)	<0.01
Coronary artery disease	16 (8.4%)	2,135 (17.5%)	<0.01
Hypertension	50 (26.3%)	4,101 (33.7%)	0.03
Cerebrovascular disease	17 (8.9%)	1,735 (14.3%)	0.04
Baseline pulmonary disease, N (%)			
Yes	44 (23.2%)	4,403 (36.2%)	<0.01
Bronchiectasis	28 (14.7%)	1,802 (14.8%)	0.98
Asthma	7 (3.7%)	1,091 (9.0%)	0.01
COPD	26 (13.7%)	2,883 (23.7%)	<0.01
Clinical symptoms, N (%)			
Respiratory failure/hypoxemia	68 (35.8%)	4,931 (40.5%)	0.19
Laboratory tests, N (%)			
Renal insufficiency	6 (3.2%)	712 (5.8%)	0.12
Liver dysfunction	42 (22.1%)	1,499 (12.3%)	<0.01
Anemia	23 (12.1%)	954 (7.8%)	0.03
Electrolyte imbalance	33 (17.4%)	1,616 (13.3%)	0.10
Cardiac insufficiency	19 (10.0%)	3,326 (27.3%)	<0.01
Hypoproteinemia	41 (21.6%)	1,651 (13.6%)	<0.01
Length of hospital stay, days, M ± SD	21.8 ± 19.5	12.7 ± 9.3	<0.01
Medical expenses, CNY, M ± SD	35,325 ± 56,871	27,818 ± 171,658	0.55

**Note:**

COPD, chronic obstructive pulmonary disease; CNY, Chinese yuan; M ± SD, mean ± standard deviation.

Subgroup analyses were performed based on sex, which showed that both the length of hospital stay and medical expenses in both male and female patients with the pulmonary abscess were much higher than those in male or female patients without the pulmonary abscess, although a statistically significant difference was only achieved for the length of hospital stay but not for the medical expenses (Table 2). Patients were also categorized into the different age groups (Table 2, based on the World Health Organization classification of 2022). The length of hospital stay for patients with the pulmonary abscess was still significantly greater than that for the patients without the pulmonary abscess in nearly each age group except the ≥90 years old group. In addition, subgroup analyses of medical expenses showed that, although the mean medical expenses in patients with the pulmonary abscess was generally higher than those in patients without the pulmonary abscess, a statistically significant difference was only achieved in patients aged under 45 years old (Table 2).

**Table 2:** Comparisons of length of hospital stay and medical expenses between patients with or without pulmonary abscess in different sex and age groups.

	Patients with pulmonary abscess (*N* = 190)	Patients without pulmonary abscess (*N* = 12,189)	*P*
Length of hospital stay, days, M ± SD	21.8 ± 19.5	12.7 ± 9.3	<0.01
Sex			
Male	23.1 ± 21.7	12.8 ± 9.6	<0.01
Female	17.8 ± 9.8	12.4 ± 8.7	<0.01
Age groups			
<45	23.0 ± 17.4	11.6 ± 8.7	<0.01
≥45, <60	19.1 ± 14.36	12.0 ± 9.4	<0.01
≥60, <75	23.3 ± 21.2	12.0 ± 8.2	<0.01
≥75, <90	22.6 ± 25.2	14.4 ± 10.3	<0.01
≥90	19.5 ± 10.6	16.9 ± 13.0	0.26
Medical expenses, CNY, M ± SD	35,325 ± 56,871	27,818 ± 171,658	0.55
Sex			
Male	39,298 ± 64,604	30,121 ± 213,232	0.61
Female	23,571 ± 17,215	23,747 ± 34,089	0.97
Age groups			
<45	39,322 ± 64,181	15,370 ± 23,527	<0.01
≥45, <60	25,826 ± 25,074	21,501 ± 34,331	0.34
≥60, <75	32,706 ± 49,289	27,534 ± 269,779	0.87
≥75, <90	50,390 ± 90,473	37,591 ± 54,307	0.19
≥90	118,762 ± 149,421	54,774 ± 102,438	0.38

**Note:**

CNY, Chinese yuan; M ± SD, mean ± standard deviation.

### Bivariate analysis of factors associated with length of hospital stay and medical expenses in patients with pulmonary abscess

**Figure 1:**
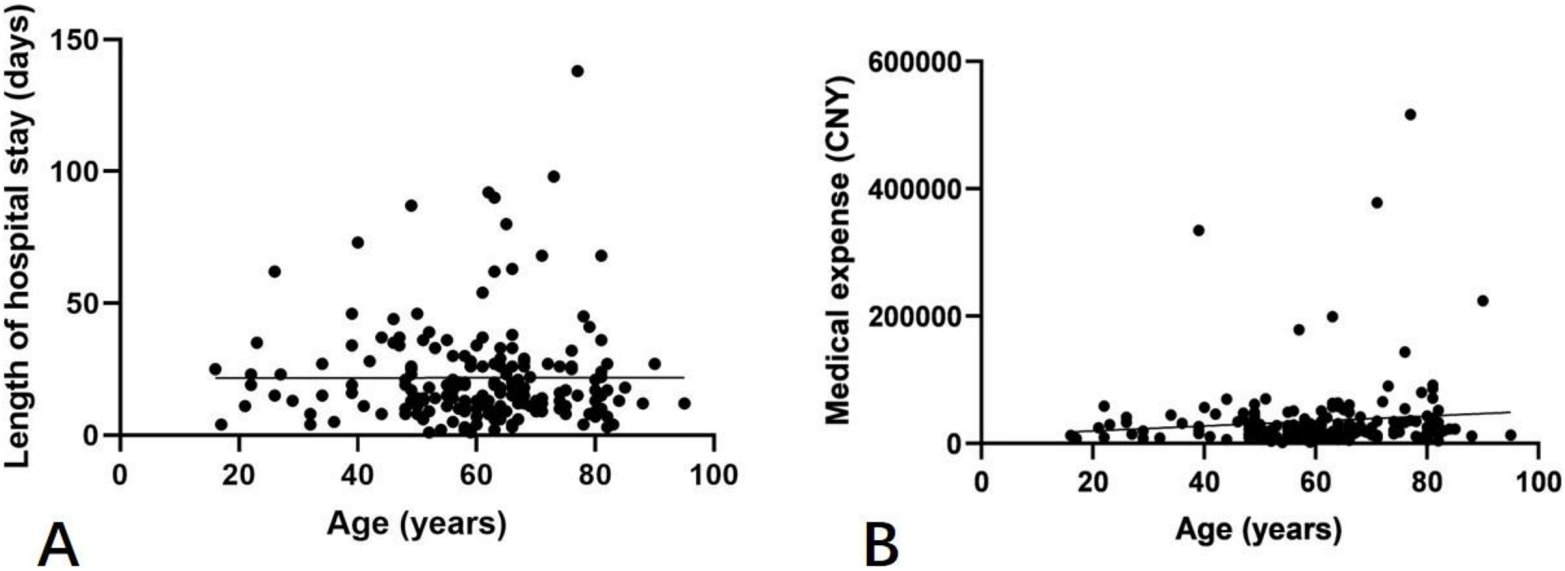
Correlations between age with the length of hospital stay (A, *r* ^2^ < 0.01, *P* = 0.99) and medical expenses (B, *r* ^2^ = 0.01, *P* = 0.15).

We further studied the factors associated with the pulmonary abscess in the hospitalized patients. There were no statistically significant associations of age with the length of hospital stay and medical expenses (Fig. 1). As shown in Tables 3 and 4, the mean length of hospital stay for male and female patients was 23.1 and 17.8 days, respectively, with a difference of 5.3 days, which was statistically significant (*P* = 0.025). The medical expenses for male patients exceeded that for female patients, and a statistically significant difference (*P* = 0.002). Patients with the baseline medical conditions, extrapulmonary disease, baseline pulmonary disease, and clinical symptoms had longer hospital stays and higher medical expenses compared to patients without these conditions. In addition, there were statistically significant differences in the length of hospital stay (*P* < 0.05) in patients with underlying extrapulmonary diseases and some abnormal laboratory test results, and in the medical expenses (*P* < 0.05) in patients with clinical symptoms and some abnormal laboratory test results (Table 3).

**Table 3:** Bivariate analysis of factors associated with length of hospital stay and medical expenses in patients with pulmonary abscess.

Variables	Length of hospital stay, days, M ± SD	*P*	Medical expenses, CNY, M ± SD	*P*
Sex		0.025		0.002
Male	23.1 ± 21.7		39,298 ± 64,604	
Female	17.8 ± 9.8		23,571 ± 17,215	
Baseline medical condition		0.54		0.44
Yes	22.5 ± 19.9		37,918 ± 54,592	
No	20.7 ± 18.9		31,435 ± 60,288	
Underlying extrapulmonary disease		0.03		0.07
Yes	25.3 ± 21.3		43,684 ± 61.892	
Diabetes	31.0 ± 25.1		50,300 ± 65,398	
Coronary artery disease	20.3 ± 22.2		31,421 ± 24,562	
Hypertension	24.0 ± 20.1		32,508 ± 30,236	
Cerebrovascular disease	25.7 ± 18.7		100,253 ± 118,543	
No	19.0 ± 17.5		28,840 ± 52,023	
Baseline pulmonary disease		0.41		0.28
Yes	19.6 ± 18.6		27,110 ± 21,325	
Bronchiectasis	22.0 ±22.1		26,551 ± 19,805	
Asthma	19.6 ± 9.9		29,552 ± 18,238	
COPD	18.2 ± 15.7		27,999 ± 20,468	
No	22.4 ± 19.7		37,800 ± 63,673	
Clinical symptoms		0.30		<0.01
Respiratory failure/hypoxemia	23.7 ± 21.3		54,416 ± 87,723	
Laboratory tests		<0.01		<0.01
Renal insufficiency	19.5 ± 8.02		57,945 ± 82,480	
Liver dysfunction	28.3 ± 23.1		47,570 ± 64,005	
Anemia	32.4 ± 32.5		77,815 ± 127,010	
Electrolyte imbalance	25.7 ± 28.1		65,582 ± 105,803	
Cardiac insufficiency	21.7 ± 14.7		67,798 ± 91,653	
Hypoproteinemia	28.1 ± 27.6		71,885 ± 108,227	

**Note:**

COPD, chronic obstructive pulmonary disease; CNY, Chinese yuan; M ± SD, mean ± standard deviation.

**Table 4:** Multivariate linear regression analysis of factors associated with length of hospital stay and medical expenses in patients with pulmonary abscess.

Variables	Length of hospital stay			Medical expenses		
	Coefficient	95% confidence interval	*P*	Coefficient	95% confidence interval	*P*
Constant	14.20	[14.81–26.93]	0.03	−11,740	[−46,829 to 23,348]	0.51
Age	−0.05	[−0.25 to 0.14]	0.58	214	[−317.6 to 745.6]	0.42
Sex	4.97	[−1.39 to 11.34]	0.12	18,242	[697.1–35,787]	0.04
Underlying extrapulmonary disease	8.58	[2.75–14.42]	<0.01	12,351	[−3,751 to 28,453]	0.13
Baseline pulmonary disease	−1.98	[−8.80 to 4.85]	0.57	−16,411	[−35,230 to 2,409]	0.09
Clinical symptoms	2.01	[−4.41 to 8.42]	0.54	18,180	[481.7–35,879]	0.04
Laboratory tests						
Renal insufficiency	−8.97	[−25.62 to 7.68]	0.29	−22,361	[−68,271 to 23,548]	0.33
Liver dysfunction	6.75	[−0.13 to 13.63]	0.05	2,231	[−16,736 to 21,199]	0.82
Anemia	12.50	[3.44–21.56]	<0.01	35,503	[10,512–60,495]	<0.01
Electrolyte imbalance	0.71	[−7.63 to 9.05]	0.87	7,872	[−15,124 to 30,868]	0.50
Cardiac insufficiency	−2.86	[−12.82 to 7.11]	0.57	18,304	[−9,174 to 45,782]	0.19
Hypoproteinemia	2.26	[−5.48 to 10.01]	0.57	23,994	[2,637–45,351]	0.03

### Multivariate linear regression analysis of factors associated with length of hospital stay and medical expenses in patients with pulmonary abscess

We performed the multivariate linear regression analysis to identify factors associated with the length of hospital stay and medical expenses in patients with pulmonary abscess (Table 4). The extrapulmonary disease and clinical symptoms were associated with the length of hospital stay and medical expenses, respectively. In addition, anemia was associated with both the length of hospital stay and medical expenses. Sex and hypoproteinemia were associated with the medical expenses.

## Discussion

In this retrospective study, we systematically analyzed the risk factors associated with the length of hospital stay and medical expenses in patients hospitalized for pulmonary abscess during the last 5 years in our hospital. The presence of a pulmonary abscess was associated with a prolonged length of hospital stay and higher medical expenses compared with the patients without pulmonary abscess. Among the patients hospitalized for the pulmonary abscess, the male sex, underlying diseases, clinical symptoms, and certain abnormal laboratory tests were associated with prolonged length of hospital stay and higher medical expenses. These patients with prolonged hospital stay and higher medical expenses might require more advanced medical care. Therefore, our study could help to guide the risk stratification of pulmonary abscess patients by utilizing the clinicopathological characteristics.

Pulmonary abscess is one of the most severe forms of pulmonary infection. Its treatment requires enhanced and prolonged antibiotic therapy. Thus, it might cause a longer hospital stay and higher medical expenses. As demonstrated in our study, patients hospitalized with the pulmonary abscess had a longer hospital stay and higher medical expenses compared to the patients with other pulmonary diseases. These results were different from a previous study, which showed no statistically significant difference in the hospital stay between patients with or without pulmonary abscess (Huang et al., 2010). The different results might be due to the small number of cases in that previous study. Our results highlighted the clinical challenges and economic burden posed by the presence of the pulmonary abscess. The management of pulmonary abscess patients can be improved as the risk factors associated with worse clinical outcomes of this disease are increasingly understood.

Interestingly, the male sex was found to be associated with higher medical expenses in the pulmonary abscess patients in both the bivariate and multivariate analyses. This result was never reported previously. One possible explanation was that the male patients usually had a long-term smoking history and a higher risk of underlying pulmonary diseases like COPD, emphysema, or bronchitis (Cai et al., 2019; Dyrhovden et al., 2019). These pre-existing conditions could compromise the pulmonary functions, which predisposed the development of pulmonary infection as well as requiring more advanced treatments with high medical costs.

The extrapulmonary disease was associated with the length of hospital stay. Common extrapulmonary underlying diseases included diabetes mellitus, coronary heart disease, cerebrovascular diseases, and renal insufficiency (Sabbula, Rammohan & Akella, 2022). These diseases might not directly result in a compromised pulmonary function. However, their existence might lead to deterioration of the patient’s general health status, with a compromised immune response in some patients. All of these could interfere with a patient’s recovery from a pulmonary abscess, as evidenced by the prolonged hospital stay in patients with underlying diseases in our study. The most common cerebrovascular disease is cerebral infarction, which is associated with severe consequences due to paralysis and long-term bed lying (Yang et al., 2020). Thus, it is more challenging to treat a pulmonary abscess among patients with pre-existing cerebrovascular diseases. These findings indicated that precaution and enhanced managements should be taken in pulmonary abscess patients with underlying diseases, especially cerebrovascular diseases.

As expected, accompanying symptoms are also critical information for risk stratification of pulmonary abscess patients. Accompanying symptoms were associated with higher medical expenses among patients with the pulmonary abscess. More severe clinical symptoms might suggest more discomforts to the patients that required more management strategies, which increased the medical expenses.

Interestingly, anemia was associated with the both the longer hospital stay and higher medical expenses in patients with pulmonary abscess. Anemia could be a sign for chronic illness and poor baseline health condition (Wiciński et al., 2020). Similar to anemia, hypoproteinemia is also an indicator for poor health condition (Keller, 2019). Hypoproteinemia could cause more treatment supports with high medical expenses. Our study was the first to highlight the clinically significant correlation between certain laboratory tests and pulmonary abscess.

Our study had several limitations. First of all, only limited clinical variables were included in the current study. Many other clinical characteristics, such as the location of the pulmonary abscess, etiological pathogens, time point of therapeutic intervention, and therapeutic strategies, could also be critical risk factors for pulmonary abscess patients and should be evaluated in a further study (Sabbula, Rammohan & Akella, 2022; Yang et al., 2020). Second, we only analyzed the length of hospital stay and medical expenses. Other outcome measurements, such as the incidence of relapse, long-term pulmonary function, and mortality, are also worthy of further study. Last but not least, our study could carry biased due to its retrospective study design in a single research center. The study sample size was also modest. Therefore, future large-scale studies are required to validate the findings of the present study.

## Conclusions

In conclusion, the mean length of hospital stay was longer in patients with the pulmonary abscess than those without the pulmonary abscess. The length of hospital stay and medical expense were associated with sex, clinical symptoms, extrapulmonary disease, and abnormal laboratory tests in patients with the pulmonary abscess. These clinical characteristics should be carefully considered to tailor the management strategy in the pulmonary abscess patients.

## Supplemental Information

10.7717/peerj.15106/supp-1 Supplemental Information 1 Raw data.

## Additional Information and Declarations

### Data Availability

The following information was supplied regarding data availability: The raw data are available in the Supplemental File.
